# Marginal Adaptation and Microbial Leakage at Conometric Prosthetic Connections for Implant-Supported Single Crowns: An In Vitro Investigation

**DOI:** 10.3390/ijms22020881

**Published:** 2021-01-17

**Authors:** Peter Gehrke, Philip Hartjen, Ralf Smeets, Martin Gosau, Ulrike Peters, Thomas Beikler, Carsten Fischer, Carolin Stolzer, Jürgen Geis-Gerstorfer, Paul Weigl, Sogand Schäfer

**Affiliations:** 1 Department of Postgraduate Education, Center for Dentistry and Oral Medicine (Carolinum), University Hospital, Goethe University Frankfurt, 60323 Frankfurt am Main, Germany; 2 Private Practice for Oral Surgery and Implant Dentistry, 67059 Ludwigshafen, Germany; 3 Department of Oral and Maxillofacial Surgery, University Medical Center Hamburg-Eppendorf, 20251 Hamburg, Germany; p.hartjen@uke.de (P.H.); r.smeets@uke.de (R.S.); m.gosau@uke.de (M.G.); c.stolzer@uke.de (C.S.); 4 Department of Oral and Maxillofacial Surgery, Division of Regenerative Orofacial Medicine, University Hospital Hamburg-Eppendorf, 20251 Hamburg, Germany; 5 Department of Periodontics, Preventive and Restorative Dentistry, University Medical Center Hamburg-Eppendorf, 20251 Hamburg, Germany; u.peters@uke.de (U.P.); t.beikler@uke.de (T.B.); 6 Dental Laboratory, Sirius Ceramics, 60528 Frankfurt am Main, Germany; fischer@sirius-ceramics.com; 7 Section Medical Materials Science and Technology, University Hospital Tuebingen, 72076 Tuebingen, Germany; geis-gerstorfer@mwt-tuebingen.de; 8 Department of Prosthodontics and Head of Department of Postgraduate Education, Center for Dentistry and Oral Medicine (Carolinum), University Hospital, Goethe University Frankfurt, 60528 Frankfurt am Main, Germany; weigl@em.uni-frankfurt.de

**Keywords:** conometric connection, conical coupling, Acuris, bacterial leakage, marginal fit, CAD/CAM crown

## Abstract

Encouraging clinical results were reported on a novel cone-in-cone coupling for the fixation of dental implant-supported crowns (Acuris, Dentsply Sirona Implants, Mölndal, Sweden). However, the presence or absence of a microgap and a potential bacterial leakage at the conometric joint has not yet been investigated. A misfit and a resulting gap between the conometric components could potentially serve as a bacterial reservoir that promotes plaque formation, which in turn may lead to inflammation of the peri-implant tissues. Thus, a two-fold study set-up was designed in order to evaluate the bidirectional translocation of bacteria along conometrically seated single crowns. On conometric abutments filled with a culture suspension of anaerobic bacteria, the corresponding titanium nitride-coated (TiN) caps were fixed by friction. Each system was sterilized and immersed in culture medium to provide an optimal environment for microbial growth. Positive and negative controls were prepared. Specimens were stored in an anaerobic workstation, and total and viable bacterial counts were determined. Every 48 h, samples were taken from the reaction tubes to inoculate blood agar plates and to isolate bacterial DNA for quantification using qrt-PCR. In addition, one Acuris test system was subjected to scanning electron microscopy (SEM) to evaluate the precision of fit of the conometric coupling and marginal crown opening. Throughout the observational period of one week, blood agar plates of the specimens showed no viable bacterial growth. qrt-PCR, likewise, yielded a result approaching zero with an amount of about 0.53 × 10^−4^ µg/mL DNA. While the luting gap/marginal opening between the TiN-cap and the ceramic crown was within the clinically acceptable range, the SEM analysis failed to identify a measurable microgap at the cone-in-cone junction. Within the limits of the in-vitro study it can be concluded that the Acuris conometric interface does not allow for bacterial translocation under non-dynamic loading conditions.

## 1. Introduction

Due to their favorable long-term clinical results, fixed implant-supported restorations are considered a valuable treatment alternative for the replacement of single or multiple teeth [1,2]. In principle, the fixation of an implant reconstruction can be achieved by either screw-retaining or cement-retaining. Apart from the personal preference of the practitioner and the relative advantages and disadvantages in the clinical application of the respective fixation technique, the type of retention might have an influence on the prosthetic aftercare of implant patients. Extensive systematic reviews of screw vs. cemented prostheses have shown that both types of fixation affect the clinical result in differing ways and that neither retention mode was distinctly more beneficial in relation to the other [3,4]. Cemented reconstructions presented more biological complications, such as crestal bone loss of more than 2 mm, due to undetected cement residue, while screw-retained restorations displayed more technical difficulties, such as loosening of the retaining screw or screw fracture. In addition to the type of fixation, the individual components, the design of the structure and the materials used may also affect the incidence of complications. In this context, systematic multivariate analyses have demonstrated the general challenge of screw-retained and cemented restorations in terms of complications, however a higher rate of technical and biological complications with cemented restorations has been observed [5].

In order to overcome the aforementioned problems associated with the attachment of fixed implant-supported restorations, the use of a conical coupling retention to support single crowns has recently been introduced [6]. In this conometric configuration, a cone-in-cone morse tapered connection between the abutment and the crown is utilized for fixation. The system consists of a conical titanium nitride-coated (TiN) cap that is cemented extraorally into an all-ceramic crown and subsequently fixed by friction on a conical abutment (Acuris Cap/Acuris Abutment, both Dentsply Sirona implants, Mölndal, Sweden). The definitive restoration is being attached to the abutment without screws or cement. The sealing and retention efficiency of the morse taper conometric system is achieved by means of a wedge effect. When an adequate insertion force is exerted, the cervical margin of the TiN coping is slightly deflected, creating elastic tension in both the coping and the abutment. The Acuris restorative concept is modified from previously published conometric approaches as the cementation procedure between the coping and the crown is performed extraorally by a dental technician. This particularity is possible due to the presence of a newly introduced antirotation function. Retention of the taper coupling is achieved only when the coping is fully seated on the abutment. The nitride coating of the cap is obtained by a plasma layering process. In this technique, titanium and nitrogen ions are combined with TiN and are molecularly linked to the titanium substrate of the coping. Studies describe TiN as biocompatible, plaque and bacteria inhibiting with chemical inertness [7,8]. Similarly, a recent review of the biological effects of different abutment materials on peri-implant bone stability found no significant bone loss around TiN abutments over time [9]. Due to its golden hue, it achieves an esthetically pleasing tone under the peri-implant mucosa, leading to a lower degree of unfavorable color shift of the soft tissues [10,11].

The employment of conically coupled abutments with different designs indicated promising prosthetic results. Hybrid-acrylic prostheses and lithium disilicate or monolithic zirconia fixed partial dentures (FPDs) and single crowns (SCs) retained by conical friction showed minimal technical problems and healthy peri-implant soft-tissues [12,13,14,15,16]. The reported benefits of this concept are the avoidance of cements or the omission of additional retaining screws, the formation of an anatomical soft tissue profile, ease of maintenance, and the use of low-cost prefabricated components.

While encouraging in-vitro and in-vivo results have been reported for cone-in-cone morse-tapered connections used to retain implant-supported FPDs and SCs [17,18,19], little information is available on the fit and risk of bacterial leakage at the abutment to restoration junction [20]. A misfit and a resulting gap between the conometric components at the level of the restoration margins could serve as a bacterial reservoir that promotes plaque formation. This in turn may lead to inflammation of the peri-implant tissues and crestal bone resorption [21]. Data on the microbiological sealing of the novel conometric Acuris connection are lacking completely. Thus, the aim of the present investigation was to examine the microbial sealing ability along the prosthetic connection of conometrically seated Acuris single crowns in vitro. The null hypothesis tested was that the conometric interface does not allow for bacterial translocation.

## 2. Results

### 2.1. Bacterial Exit Out of the System

**Figure 1:**
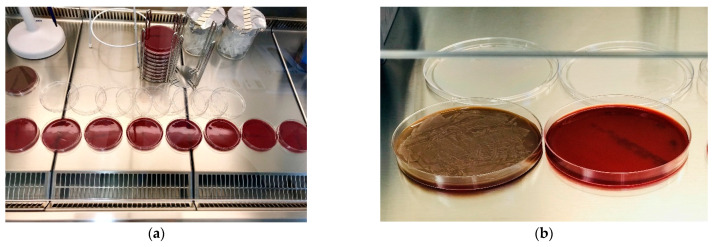
(**a**) Inoculated blood agar plates with specimens from implants 1–8 on test day one. All implants show no signs of bacterial growth similar to negative control. Inoculated blood agar plates with positive control (left) and negative control (right). (**b**) Positive control shows clear signs of bacterial colonization, turbidity and hemolysis, whereas negative control shows no signs of bacterial growth.

**Figure 2:**
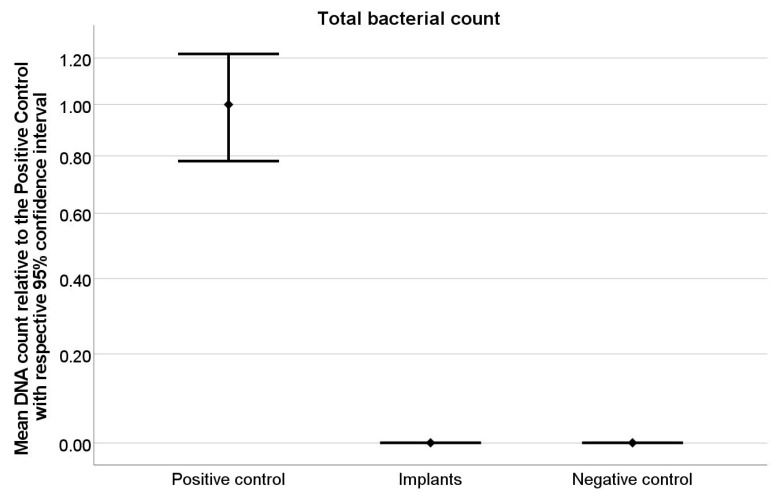
Graphical illustration of the statistical results in Table 2, which is based on the statistical qrt-PCR amplification results for positive controls (*n* = 4—four amplification values per positive control), negative controls (*n* = 4—four amplification values per positive control) and implant specimens (*n* = 16—two amplification values per implant). A significant difference of qrt-PCR results between the positive control and test groups (*p* < 0.001) and no difference between the negative control and test groups (*p* = 0.99) could be shown.

The negative control, blood agar plates of the specimens showed no viable bacterial growth by turbidity, colonization or hemolysis (Figure 1a). In contrast, the positive control displayed an obvious bacterial growth (Figure 1b). qrt-PCR, likewise, yielded a result approaching zero with an amount of about 0.53 × 10^−4^ µg/mL DNA for the specimens and 2.65 µg/mL DNA for the positive control (Table 1). Statistical analysis confirmed the qrt-PCR results indicating a significant difference between positive control and test specimens (*p* < 0.001) (Table 2, Figure 2), whereas, no difference was found between negative control and specimens (*p* = 0.99). Possible deviations for the test days caused no significant difference on the mean bacterial count (*p* = 0.69) (Table 3).

### 2.2. Bacterial Entry into the System

All specific primers tested in qrt-PCR revealed no bacterial DNA for the specimens, in contrast to the positive control. Due to an inadequate seating of the TiN cap on the abutment of specimen No. 6, only poor conometric coupling could be achieved resulting in an additional positive control with an increased amount of DNA.

### 2.3. Scanning Electron Microscopy

**Figure 3:**
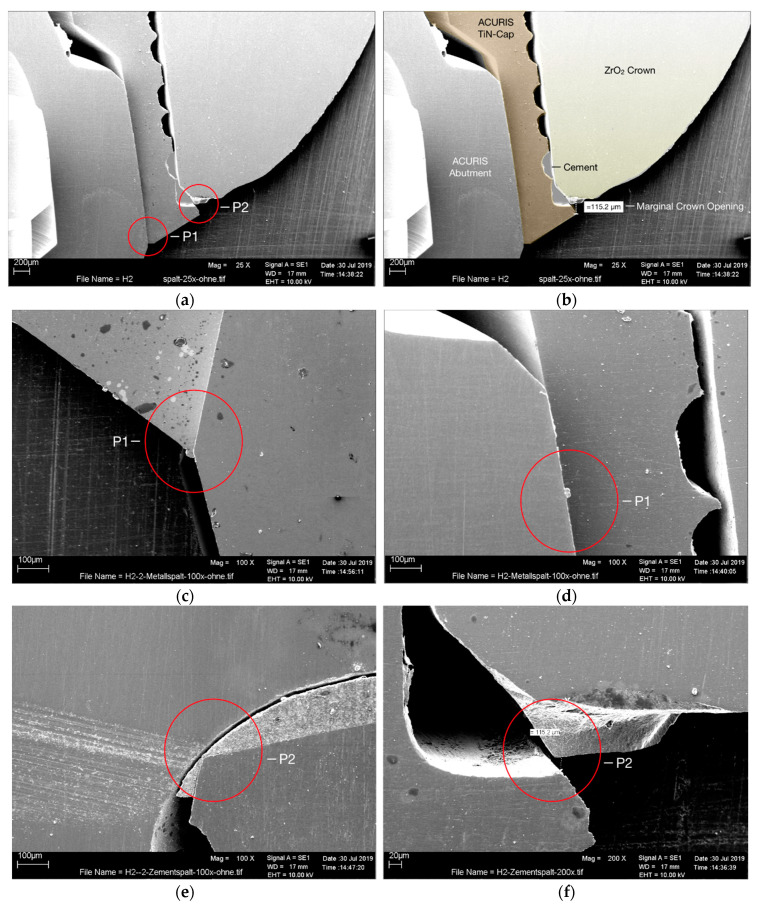
SEM images (**a**–**f**) of morse taper Acuris coupling (measuring points P1) and marginal opening of zirconia crown (measuring points P2) at different magnifications (25×, 100× and 200×). SEM images **c** and **d** (100×) display no measurable microgap at the cone-in-cone Acuris junction (P1). Pictures e and f identified a luting gap/marginal opening between the Acuris TiN-cap and the ceramic crown within the clinically acceptable range of approximately 115 μm (P2).

While the luting gap/marginal opening between the TiN-cap and the ceramic crown (P2) was within the clinically acceptable range of approximately 115 μm, the SEM analysis failed to identify a measurable microgap at the cone-in-cone Acuris junction (P1) (Figure 3a–f).

## 3. Discussion

A dental implant system comprises the endosseous portion and connects to a superstructure that ultimately restores function and esthetics. Although both are intimately joined in one-piece implants, for two-part implant systems, a microgap at the interface between implant and abutment and at the interface between abutment and prosthesis seems unavoidable. The latter include a removable transmucosal abutment that is attached on one side to the implant and on the other side to the restoration. As a result, an implant–abutment interface and an abutment–restoration interface are generated. The size of the latter depends on the respective manufacturer and seems to be limited to less than 50 μm for current implant systems [13]. Larger microgaps at the abutment–prosthesis interface may be expected, as the restorative part is usually not prefabricated and may therefore present an inferior fit. Bacterial pathways evolve along the interfaces progressing to the internal implant and prosthesis. Microorganisms can migrate into these interfacial microgaps forming a bacterial reservoir and, when located closely to the bone, can play a role in the development of peri-implant inflammation and subsequent bone loss [21,22]. Thus, marginal and internal adaptation at the abutment–restoration interface are significant factors as they are directly related to biological integrity, structural rigidity and maintenance of peri-implant tissue health. Furthermore, under load conditions, both microgaps can be widened further [19]. In this context, it could be demonstrated that the geometry of the implant–abutment connection can have an influence on the extent of microbial penetration and that implants with internal morse taper connection exhibit a lower contamination level compared to other geometries [22,23]. 

While encouraging clinical results have been reported for cone-in-cone morse-tapered connections at the implant abutment junction [17], little information is available on the fit and risk of bacterial leakage at the morse taper abutment to restoration junction [20]. The primary objective of the current in vitro study was therefore to evaluate the microbial sealing ability along the prosthetic connection of conometrically seated Acuris single crowns in vitro. Bacterial leakage from the conometric coupling surfaces and the respective proliferation were monitored in the present study every 48 h for one week. Observation periods exceeding one week should be avoided due to the associated risk of obtaining false-negative observations [24]. In comparison to microgaps at the implant abutment junction [25], larger gaps are commonly expected at the restorative interface, since the required prosthetic components are individually manufactured and processed in the dental laboratory. In cemented restorations, a considerable marginal discrepancy increases the thickness of the cement exposed to the oral fluids, resulting in the dissolution of cement and marginal leakage. Moreover, the internal crown adaptation has an influence on the long-term stability of an all-ceramic crown. An interrelation correlation between increased cement thickness and reduced flexural strength of ceramics has been proven. It has been reported that marginal openings of metal-ceramic crowns ranging from 50 to 120 μm are clinically acceptable [26,27,28]. For CAD/CAM all-ceramic crowns, the acceptable marginal gap is reported to be less than 70 μm [29,30]. However, there is no consensus in the literature on the limits of clinically acceptable marginal adaptation, and a maximum deviation of 120 µm, as defined by McLean in 1971, is still widely used [27]. The diversity of study results may be related to the material used for the restorations, the method of measurement, the sample size, the demographic composition of the surveys and the type of review.

An improved seal in terms of microbial leakage may be achieved by using prefabricated components. The recently introduced Acuris conometric coupling examined in the current in-vitro study uses the friction between the abutment and a prefabricated titanium nitride (TiN) coping for retaining a crown. A full-ceramic crown is luted extraorally onto this prefabricated final TiN coping and then attached intraorally to the abutment to provide a conometric friction retention [6]. The final coping is indexed to match the corresponding antirotational connection on the top of the abutment by using an axially directed force from a calibrated striker (Conometric fixation tool, Dentsply Sirona Implants, Mölndal, Sweden). This allows for correct seating and orientation of the crown and avoids rotation. The Acuris coupling is thus a fixed retention, but can be retrieved by the dentist for maintenance. Since the primary aim of the present investigation was to examine the potential bacterial leakage at the TiN coping–abutment interface, the possible bacteria-inhibiting influence of titanium nitride as a biocompatible coping material was not addressed in the study.

The specific qrt-PCR analysis and agar plate spreading of the present in vitro investigation revealed no bacterial transfer from and into the prosthetic connection of conometrically seated Acuris single crown-units. The null hypothesis that the conometric interface does not allow for bacterial translocation can thus be considered as accepted. Comparison of the present findings can only be made with caution, as the available literature data are limited, and bacterial translocation appears to be dependent on the particular geometry and accuracy of the prefabricated components of each system. The results of a recent in vitro study with two other conometric systems using different materials showed a minimal microbial concentration of less than 1 × 10^2^ copies/μL in real-time PCR for all positive assemblies (10 of 20 total assemblies) [20].

It has been suggested that the sealing performance of conometric morse taper systems and their retention capacity is achieved by the wedge effect. Conical coupling retention is, however, only achieved when the coping is fully seated on the abutment. During the in vitro study-setup, very little room for movement was available in the anaerobic chamber, which resulted in suboptimal conditions for the assembly of the TiN copings. As a result, one coping of implant sample No. 6 was incorrectly retained and consequently showed increased DNA values. It is important to note that this increase was clearly due to inappropriate handling of the TiN cap rather than a system relevant bacterial leakage. This sample was therefore only used as an additional positive control. In a clinical scenario, incomplete seating of the TiN coping-crown unit would result in retention deficiency and instant crown detachment. The seating of the coupling is thus checked immediately after insertion by means of a periapical radiograph. Tight proximal contacts or excessive submucosal emergence profiles may be other reasons for inadequate crown fit and poor retention in a clinical scenario. A drawback of the present in-vitro study is the relatively small number of samples, which should be expanded in future investigations. A further limitation relates to the fact that the contribution of loading on bacterial leakage at the conometric interface was not evaluated. It has been shown that testing of the implant–abutment connection under dynamic-loading conditions is an important part of the experimental design to evaluate the bacterial colonization of dental implants [19,31]. The same can be assumed for the restorative abutment junction. In addition, extrapolation of in vitro results to in vivo conditions should be performed with caution. Only the outcome of long-term clinical trials on periodontal health will allow for a classification of relevance of the obtained in vitro results. The existing clinical results of conometrically fixed partial (FDP) and complete prosthesis (CP) using the same friction principle, demonstrated minimal technical and biological complications within a 2- and 5-year period, respectively [15,32]. Similar favorable clinical results can be assumed for the single-tooth replacement with Acuris. Further studies are required to determine marginal adaptation and microbial leakage in conometrically retained single crowns.

Referring back to our hypothesis, the microbial sealing ability of the conical prosthetic connection was evaluated in a double-verification study set-up. No bacterial translocation was detected, neither from nor into the Acuris abutment system. Amounts of both living and dead bacteria were measured using agar plate spreading and qrt-PCR. Agar palates exhibited no bacterial growth for the implant specimens according to the negative control. Results of qrt-PCR measurement for the bacterial exit out of the system confirmed no significant difference between the implant specimens and the negative control (*p* < 0.001). Hence, a holistic examination of bacterial colonization for the cone-in-cone coupling showed no microbial leakage. SEM analysis revealed a marginal luting gap between the Acuris TiN-cap and ceramic crown within a clinically acceptable range of 115 μm, but no microgap at the cone-in-cone Acuris junction. A further study is planned to expand comparability and to reveal clinical implications for practitioners. We included the four most prevalent oral microbes in our bacterial mixed culture solution used for bacterial entry and exit out of the implant system (*Streptococcus mutans*, *Actinomyces naeslundii, Fusobacterium nucleatum* and *Porphyromonas gingivalis*). These bacteria are usually benign—however, under certain conditions, they may cause caries, parodontitis, mucositis or perimplantitis [33]. Bacterial culturing conditions were chosen according to the German Collection of Microorganism and Cell Cultures (Leibniz Institute DSMZ, Braunschweig, Germany). The testing period of one week was chosen to cover long time periods while still preventing false negative results by replacing the culture medium every 48 h with fresh medium to provide enough bacterial growth space. This issue has been previously discussed in other studies (19). The qrt-PCR results confirmed the adequacy of the test conditions, as a positive bacterial count was determined for each positive control on each test day.

## 4. Materials and Methods

### 4.1. General Study Set-Up

**Figure 4:**
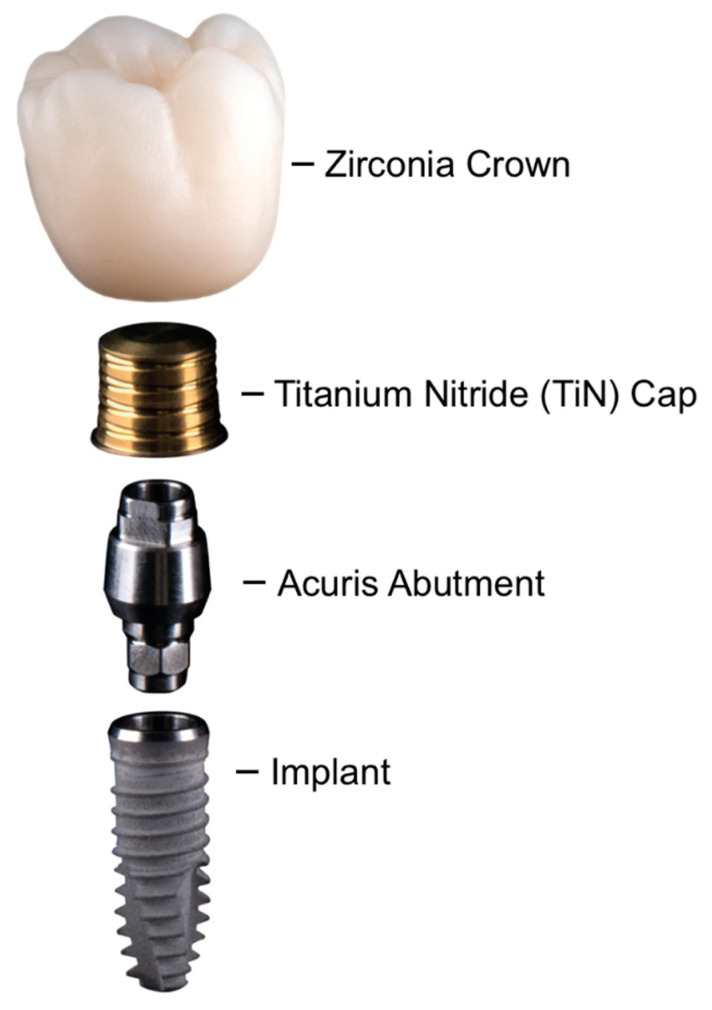
Schematic diagram of the Acuris conometric concept with the Xive implant system.

A two-fold study set-up was designed in order to evaluate the bidirectional translocation of bacteria through the sealed conometric system. A total of 10 conometric abutments (Acuris, A0, GH 3 mm, Dentsply Sirona Implants, Mölndal, Sweden) were joined to screw implants (Xive, D 3.8/L 13 mm, Dentsply Sirona Implants, Mölndal, Sweden) with a new titanium abutment screw and torqued to 24 Ncm, as recommended by the manufacturer (Figure 4). The experimental setup proceeded as follows: The sterile delivered original implants were unpacked and the corresponding Acuris abutments were connected to the implants. The connection between the implant and abutment consisted of an internal hex interface. The abutments were then screwed to the implants via a retaining screw using a precalibrated manual torque wrench. In the Acuris system, both the TiN coping matrix and the patrix of the abutment have an antirotational feature. The TiN caps were thereafter manually seated to the antirotation portion of the abutments. The friction fit was achieved by applying an axially directed force from a dedicated fixation tool with a calibrated striker (Conometric fixation tool; Dentsply Sirona Implants, Mölndal, Sweden). Resembling a clinical setting, the ultimate fixation of the TiN copings was checked visually and by manual non-calibrated pull-off tests. Each system was subsequently subjected to autoclaving (AUTOCLAVE SYSTEC V-40, Systec GmBH, Linden, Germany). All the following work steps took place under anaerobic conditions at 37 °C in a Whitley A35 Workstation (Whitley A35 Workstation Don Whitley Scientific, Bingley, United Kingdom). For both approaches of examining bidirectional translocation, a bacterial mix culture suspension comprised of anaerobic early colonizing *Streptococcus mutans* (*Streptococcus mutans*, DSM 20523, German Collection of Microorganisms and Cell Cultures GmbH, Leibnitz, Germany), moderate colonizing *Actinomyces naeslundii* (*Actinomyces naeslundii,* DSM 17233, German Collection of Microorganisms and Cell Cultures GmbH, Leibnitz, Germany), *Fusobacterium nucleatum* (*Fusobacterium nucleatum*, DSM 15643, German Collection of Microorganisms and Cell Cultures GmbH, Leibnitz, Germany) and late colonizing *Porphyromonas gingivalis* (*Porphyromonas gingivalis*, DSM 20709, German Collection of Microorganisms and Cell Cultures GmbH, Leibnitz, Germany) strains was prepared.

### 4.2. Bacterial Exit Out of the System 

Examining bacterial leakage out of the implant system, eight implants (*n* = 8) were used as test specimens, one implant was used as the negative control (*n* = 1), and for the positive control, 4 µL pure bacterial mixed culture solution was utilized (*n* = 1). One of the total ten implant systems was previously used for a pretest and could not be included in this set-up. The occlusal opening of eight Acuris abutments that were previously mounted on the respective implants was filled with 4 µL of bacterial mixed culture of anaerobes. Corresponding TiN-caps were seated on the abutments and fixed according to the manufacturer’s recommendation (Figure 4). The implants were sanitized with 70% ethanol (EtOH) and placed in sterile 1.5 mL Eppendorf tubes, which were filled with 1 mL bacterial culture medium (CDC) in order to provide an optimal environment for bacterial colonization. As a positive control, 4 µL bacterial mixed culture were directly filled into an Eppendorf tube and as a negative control, an Acuris abutment was filled with 4 µL bacterial culture medium (CDC) instead of the bacterial mixed culture. After an incubation period of 48, 96, 144 and 192 h 100 µL sample was taken from each Eppendorf tube for the total and viable bacterial count analysis: 50 µL to inoculate blood agar plates and 50 µL to process with a DNA Isolation Kit (innuPREP DNA Isolation Kit, Analytik Jena AG, Jena, Germany) in order to quantify the DNA amount with qrt-PCR (quantitative Real-Time-PCR, CFX96 Touch Real-Time PCR Detection System, Bio-Rad Laboratories, Berkeley, California, USA). PCR (Polymerase Chain Reaction) run was performed utilizing universal eubacterial 16S-rRNA primer (HDA1 GACTCCTACGGGAGGCAGCAGT, E1115R AGGGTTGCGCTCGTTGCGGG).

### 4.3. Bacterial Entry into the System

In order to confirm the bacterial exit results, specimens were tested for bacterial leakage into the conometric system. In this test set-up, nine implant specimens were tested (*n* = 9), one implant was used as a negative control (*n* = 1), and for the positive control, 4 µL pure bacterial mixed culture solution was utilized (*n* = 1). For this purpose, after completion of the first part of the study design, all implant–abutment conometric samples were decontaminated using a DNA AWAY Kit for surface and device decontaminations (Thermo Scientific Molecular BioProducts DNA AWAY Kit, Analytik Jena AG, Jena, Germany). The process was followed by autoclave sterilization. Cleaning success was controlled with qrt-PCR.

Of the originating bacterial mixed culture solution 30 mL was separated and filled into a new reaction tube. Nine Acuris abutments were occlusally filled with 4 µL culture medium to ensure an optimal environment for bacterial colonization and conometrically fixed with Acuris TiN-caps (Figure 4). The tenth abutment was the negative control filled with 4 µL bacterial culture solution. All nine conometrically sealed implant–abutment specimens were placed into the bacterial mixed culture solution; the negative control was stored separately in a reaction tube with culture medium only. For one week, every 48, 96, 144 and 192 h, 20 mL mixed culture solution was eliminated from the original reaction tube and replaced with fresh bacterial culture medium to provide space and fresh nutritive for bacterial growth. At the same time, two implants were removed from the reaction tube, washed with phosphate buffered saline (PBS), sanitized with 70% ethyl alcohol (EtOH) and the conometric abutment seal was opened by carefully removing the TiN-cap. The solution inside was processed with a deoxyribonucleic acid (DNA) Isolation Kit (innuPREP DNA Isolation Kit, Analytik Jena AG, Jena, Germany) and the specific amount of DNA was quantified with qrt-PCR utilizing specific primers for the examined bacterial strains [34] (Table 4).

### 4.4. Scanning Electron Microscopy

Furthermore, an additional randomly selected Acuris test system (Acuris abutment, A0/ GH 3 mm on Xive implant D 3.8/L 13 mm, Dentsply Sirona Implants, Mölndal, Sweden) was subject to scanning electron microscopy (SEM, scanning electron microscope LEO 1430, Zeiss, Jena, Germany). Both, the accuracy of fit of the morse taper coupling and the marginal opening of a monolithic zirconia crown (Zirlux, Henry Schein, Langen, Germany) that had been cemented extraorally on the Acuris TiN-Cap before its conometrical seating, were analyzed microscopically. The measurements were determined at two predefined reference points (P1 = morse taper coupling and P2 = marginal opening) according to the characteristic design of the components (Figure 4). The Acuris test specimen was embedded in a polyurethane-based resin (Sherapolan 2:1, Shera Werkstofftechnologie, Lemförde, Germany) in a standardized manner with specimen grips (UNICLIP, Wirtz/Bühler, Esslingen, Germany). The horizontal alignment and the precutting to the desired sample dimensions were accomplished in a fully automated process using a precision grinding and cutting device (Accutom-50, Struers, Willich, Germany). After adjusting the required parameters (accuracy ±5 µm, cut-off wheel width 0.6 mm), polished thin sections were generated under water cooling and continuous monitoring for macro- and microscopic integrity (25× magnification, Photomacroscope, Wild, Heerbrugg, Switzerland). Upon final inspection, the sample was sputtered with Au-Pd for SEM evaluation.

### 4.5. Statistical Analysis

Statistical analysis was conducted using SAS 7.4 (SAS Institute Inc., Cary, North Carolina, USA). The mean value of qrt-PCR amplification results for positive controls (*n* = 4—four amplification values per positive control), negative controls (*n* = 4—four amplification values per positive control) and implant specimens (*n* = 16—two amplification values per implant) were used to calculate mean bacterial DNA amount in mg/mL (Table 1) counts. The latter were compared using an exponential-linear model*. p*-values < 0.05 were considered statistically significant.

## 5. Conclusions

Within the limits of the in-vitro study it could be concluded that the Acuris conometric interface did not allow for bacterial translocation under non-dynamic loading conditions. While the luting gap between the prefabricated TiN-cap and the ceramic crown was within the clinically acceptable range, no microgap could be detected at the cone-in-cone Acuris junction by SEM analysis.

## Figures and Tables

**Table 1:** Bacterial exit out of the system. Mean DNA amounts in mg/mL of the implant specimens in comparison to mean positive and mean negative control on all test days measured by the qrt-PCR amplification results for positive controls (*n* = 4—four amplification values per positive control), negative controls (*n* = 4—four amplification values per positive control) and implant specimens (*n* = 16—two amplification values per implant).

Specimens	Day 1 mg/mL	Day 2 mg/mL	Day 3 mg/mL	Day 4 mg/mL
Positive Control	2.812127	1.35693	0.540473	0.216807
Implant 1	0.000572	0.000623	0.000709	0.000643
Implant 2	0.000638	0.00062	0.000633	0.000611
Implant 3	0.000598	0.000616	0.000648	0.000671
Implant 4	0.000587	0.000634	0.000657	0.000657
Implant 5	0.000592	0.000634	0.000675	0.000625
Implant 6	0.000583	0.000664	0.000629	0.00061
Implant 7	0.000633	0.000622	0.000684	0.000622
Implant 8	0.000643	0.000644	0.000609	0.000653
Negative Control	0.000646	0.000613	0.000642	0.000638

**Table 2:** Statistical analysis of qrt-PCR amplification results for positive controls (*n* = 4—four amplification values per positive control), negative controls (*n* = 4—four amplification values per positive control) and implant specimens (*n* = 16—two amplification values per implant). Comparison of mean DNA values in mg/mL (Table 1) of the control and test groups demonstrated a significant difference of qrt-PCR results between positive control and test groups (*p* < 0.001), whereas no difference was found between the negative control and test groups (*p* = 0.99).

Differences of Groups/Least Squares Means									
Group	Group	Estimation	Standard Error	DF	t-Wert	Pr > |t|	Alpha	Lower	Upper
Positive Control	Negative Control	7.5188	0.7192	36	10.45	<0.0001	0.05	6.0602	8.9775
Positive Control	Test specimens	7.5211	0.5471	36	13.75	<0.0001	0.05	6.4115	8.6307
Negative Control	Test specimens	0.002268	0.5303	36	0.00	0.9966	0.05	−1.0733	1.0778

**Table 3:** While the comparison of test and control group had a significant effect on the results of bacterial growth (*p* < 0.001), statistical analysis revealed no significant difference for the mean bacterial count at different test days (*p* = 0.69).

Type III Test of Effects				
Effect	No. DF	Den DF	F-Value	Pr > F
Day	1	36	0.16	0.6910
Group	2	36	95.62	<0.0001

**Table 4:** Specific primer sequences for qrt-PCR and references of their applicability.

Organism	Primer	Primer Sequence	Reference of Primer Applicability
*Porphyromonas*	CA-PG-F/R	AGGCAGCTTGCCATACTGCG	Carrouel F. et al.,
*Gingivalis*		ACTGTTAGCAACTACCGATGT	2016 [34].
*Streptococcus*	MKD-FV/RV	GGCACCACAACATTGGGAAGCTCAG	Hoshino T. et al.,
*Mutans*		GGAATGGCCGCTAAGTCAACAGG	2004 [35].
*Actinomyces*	ACT-174-F	GGTCTCTGGGCCGTTACTGA	Ellerbrock B.,
Species	ACT-281-R	GRCCCCCCACACCTAGTG	2010 [UKD].
*Fusobacterium*	CA-FN-F/R	AGAGTTTGATCCTGGCTCAG	Carrouel F. et al.,
*nucleatum*		GTCATCGTGCACACAGAATTGCTG	2016 [34].

## Data Availability

The datasets used and analyzed during the current study are available from the corresponding author on reasonable request.
